# Anomalously strong two-electron one-photon X-ray decay transitions in CO caused by avoided crossing

**DOI:** 10.1038/srep20947

**Published:** 2016-02-10

**Authors:** Rafael C. Couto, Marco Guarise, Alessandro Nicolaou, Nicolas Jaouen, Gheorghe S. Chiuzbăian, Jan Lüning, Victor Ekholm, Jan-Erik Rubensson, Conny Såthe, Franz Hennies, Victor Kimberg, Freddy F. Guimarães, Hans Agren, Faris Gel’mukhanov, Loïc Journel, Marc Simon

**Affiliations:** 1 Theoretical Chemistry & Biology, School of Biotechnology, Royal Institute of Technology, S-106 91 Stockholm, Sweden; 2 Instituto de Química, Universidade Federal Goiás, Campus Samambaia, CP 131, 74001-970 Goiânia, Goiás, Brazil; 3 Laboratorio Nacional Luz Sincrotron, 10000 Campinas, Brazil; 4 Sorbonne Universités, UPMC Univ Paris 6, UMR7614, Laboratoire de Chimie Physique - Matière et Rayonnement, F-75005 Paris, France; 5 Synchrotron SOLEIL, l’Orme des Merisiers, Saint-Aubin, BP 48, 91192 Gif-sur-Yvette Cedex, France; 6 Department of Physics and Astronomy, Uppsala University, Box 516, 751 20 Uppsala, Sweden; 7 MAX IV Laboratory, Lund University, Box 118, 221 00 Lund, Sweden

## Abstract

The unique opportunity to study and control electron-nuclear quantum dynamics in coupled potentials offered by the resonant inelastic X-ray scattering (RIXS) technique is utilized to unravel an anomalously strong two-electron one-photon transition from core-excited to Rydberg final states in the CO molecule. High-resolution RIXS measurements of CO in the energy region of 12–14 eV are presented and analyzed by means of quantum simulations using the wave packet propagation formalism and *ab initio* calculations of potential energy curves and transition dipole moments. The very good overall agreement between the experimental results and the theoretical predictions allows an in-depth interpretation of the salient spectral features in terms of Coulomb mixing of “dark” with “bright” final states leading to an effective two-electron one-photon transition. The present work illustrates that the improved spectral resolution of RIXS spectra achievable today may call for more advanced theories than what has been used in the past.

The photophysical properties of matter are defined by the rates of radiative transitions. Since the interaction with the electromagnetic field is governed by an one-electron operator, the contribution of two-electron one-photon (TEOP) transitions can usually be ignored in comparison to one-electron transitions. In line with this TEOP, transition moments are strictly equal to zero within an one-particle Hartree-Fock approximation of the participating wave functions. On the other hand, a case of a weak TEOP transition is photoelectron “shake-up” which is governed by the sudden creation of a core hole potential where the ejected core photoelectron is accompanied by valence excitations^1^,^2^,^3^,^4^. TEOP transitions to “dark” states, opened by this electron correlation effect, were early observed as weak high-energy satellites lines in X-ray photoelectron spectra^5^. The related fundamental physical effect is the autoionization of two-electron excited states, which results in the Fano profile of the VUV absorption.^6^. In molecules, electrons are correlated not only with the motion of other electrons but also with the motion of the atomic nuclei. The motion of heavy nuclei and light electrons are commonly separated as expressed by the Born-Oppenheimer approximation (BO)^7^,^8^,^9^ with the main assumption that the lighter electrons adjust adiabatically to the motion of the heavier nuclei. However, the BO approximation is frequently broken near crossings of the potential energy surfaces of different electronic states^7^,^8^,^9^. In such cases the “dark” electronic state (*ψ*_*d*_(*r*)) can be reached by TEOP transitions through mixing with a “bright” state (*ψ*_*b*_(*r*)), via the nuclear wave functions (*χ*_*d*_(*R*, *t*) and *χ*_*b*_(*R*, *t*)):


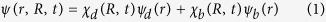


resulting in coupled non-adiabatic electronic-nuclear dynamics involving the potential surfaces of the diabatic “dark” and “bright” states. Due to the coupling of electronic wave functions through the Coulomb interelectron interaction


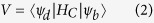


the nuclear wave packets can be written


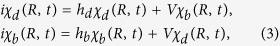


where *h*_*d*_ and *h*_*b*_ are the nuclear Hamiltonians of the “dark” and the “bright” electronic states, respectively. One should stress that in contrast to atoms the Coulomb mixing of molecular states is drastically enhanced near the crossing of the potential energy curves. The coupled equations (3) govern the electron-nuclear motion strictly and constitute a fully time-dependent approach beyond the BO approximation. Fast insight into the problem can be reached using adiabatic approximation^7^,^8^,^10^,^11^, by neglecting the kinetic energy operator in nuclear Hamiltonians. In this case the solution of the two-states eigenvalue problem (Eq. 3) is straightforward and explains the mixing of the “bright” and “dark” states. This results in adiabatic potential energy curves where the level crossing is avoided with increase of the strength of the coupling (Eq. 2). When *V* = 0 the “dark” state is not populated via radiative decay of an excited state *ψ*_*c*_ → *ψ*_*d*_ since the dipole moment of the transition is equal to zero, in contrast to the dipole moment of the transition to the “bright” state:


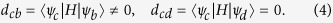


However, the “dark” state is reachable when *V* ≠ 0 due to Coulomb mixing of the states. The mixing coefficients as well as the adiabatic transition dipole moments are now sharp functions of internuclear distance *R*^8^,^12^. This is typical non-Franck-Condon effect^8^, which includes in general many different phenomena^13^,^14^. The mentioned sharp *R* dependence leads to worse numerical convergence in the adiabatic representation, as compared to the diabatic one. In spite of that the two representations provide the same final results^8^,^10^,^11^, we use the diabatic representation (3), which is better from the computational point of view^10^,^11^.

Here we study the TEOP transitions induced by the Coulomb mixing enhanced near the avoided crossing using resonant inelastic X-ray scattering (RIXS) spectroscopy (see Fig. 1). The RIXS technique gives a unique opportunity to control the quantum dynamics in coupled potentials. In RIXS, the final states of the neutral molecule are populated in a second-order process via intermediate core excited state. In a two-step picture, an incoming X-ray photon with the frequency *ω* promotes the molecule from the ground to a core excited state *ψ*_0_ → *ψ*_*c*_. The core-excitation is followed by a one-electron one-photon (OEOP) transition (with frequency *ω*′) to the dipole allowed “bright” final state *ψ*_*c*_ → *ψ*_*b*_ (Fig. 1). The initial conditions for Eq. 3 in the case of RIXS are^15^,^16^,^17^,^18^


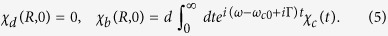


Here 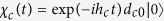 is the nuclear wave packet of core excited state coupled with the ground state by the transition dipole moment *d*_*c*0_. For the same reason, the RIXS cross section


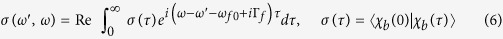


is defined only by the wave packet of the “bright” state according to the expression for the autocorrelation function *σ*(*τ*).

Although the “dark” state is not populated directly by the radiative decay (*d*_*cd*_ = 0), the Coulomb coupling (Eq. 3) of *χ*_*b*_(*R*, *t*) and *χ*_*d*_(*R*, *t*) leads to a spectral feature of *χ*_*b*_(*R*, *t*) in the energy region of the “dark” state. Via the coupling (Eq. 2), the forbidden TEOP channel *ψ*_*c*_ → *ψ*_*d*_ borrows intensity from the allowed OEOP transition *ψ*_*c*_ → *ψ*_*b*_. The effect resembles the opening of symmetry forbidden RIXS channels in polyatomic molecules, where electronic states of different symmetry are mixed by symmetry breaking in the course of asymmetric vibrations^9^,^19^,^20^. In contrast to this phenomenon, the effect studied here can occur in both diatomic and polyatomic molecules.

## Results and Discussion

We use the CO molecule as an object for studying a possible TEOP effect, as it is sufficiently small to allow necessary theoretical analysis while still bearing sufficient complexity to represent the problem. Experimental high-resolution RIXS spectra excited near the O 1s → 2*π* resonance of CO (Fig. 2) using circularly polarized X-rays are compared to predicitons in Fig. 3. The potential energy curves of the states involved in the RIXS process (Fig. 2), along with transition dipole moments between them are based on state-of-the-art *ab initio* theory. The coupled electron-nuclear dynamics was described by quantum simulations using the wave packet propagation formalism, as outlined above.

**Figure 1:**
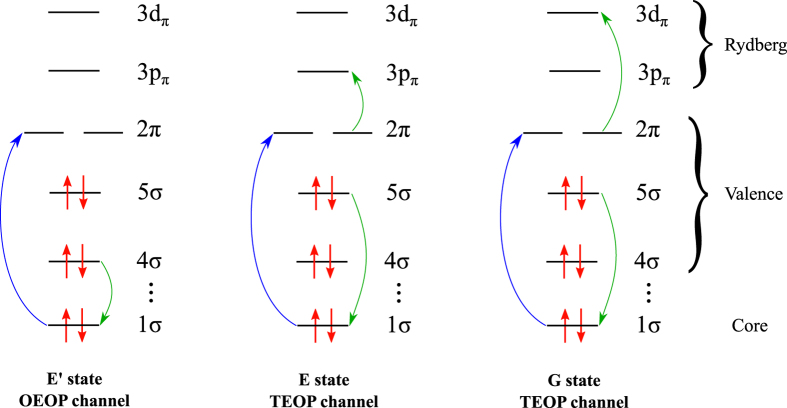
The one-electron one photon (OEOP) (left) and two-electron one photon (TEOP) (mid, right) decay transitions in RIXS of CO near the 1*σ* → 2*π* core excited state, leading to the *E*′, *E* and *G* final states, respectively.

**Figure 2:**
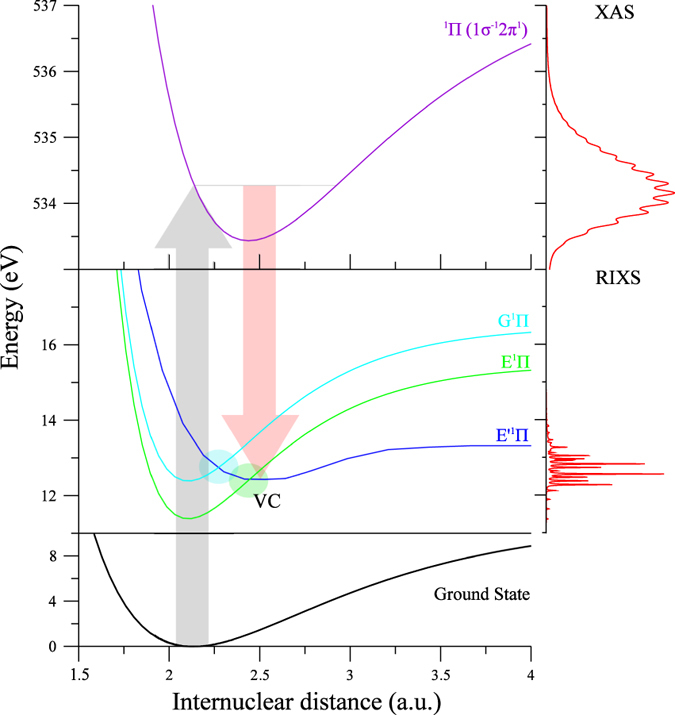
Potential energy curves of the ground, core-excited and final states. The X-ray absorption (XAS) and RIXS spectra are shown at the right panel. Regions of strong Coulomb coupling between the “bright” and “dark” states are shown schematically by shaded areas.

**Figure 3:**
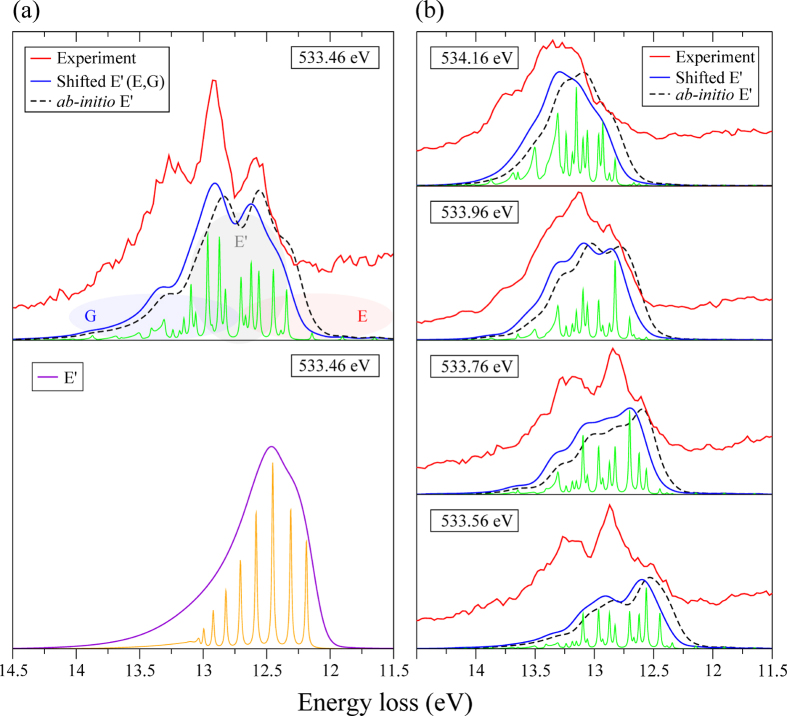
The experimental RIXS spectra (red lines) are compared with theoretical simulations using original *ab initio* PEC of *E*′ state (dashed lines) and a shifted *E*′ potential (blue lines) with *E*_min_ = 12.52 eV. The theoretical spectra are convoluted with instrumental broadening 0.16 eV. The high resolution theoretical RIXS profiles are shown below the convoluted spectra. The experimental lines are shifted slightly upwards for the sake of clarity. Panel (**a**) compares theoretical simulations for *ω* = 533.46 eV when Coulomb mixing of the crossing “bright” and “dark” states is included (upper plot) with the case when this mixing is neglected (lower plot). Spectral regions related to the valence and Rydberg states are pointed schematically by shaded areas in the plot (**a**).

**Figure 4:**
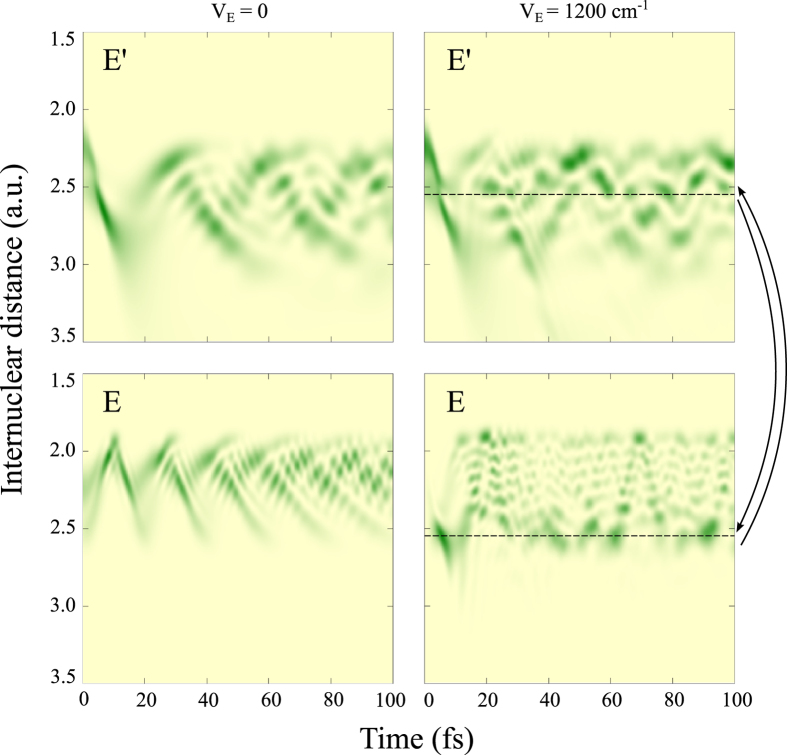
Dynamics of the vibrational wave packet in the “bright” *E*′ (upper panels) and “dark” *E* states (lower panels). Here, the left panels show the case when coupling (Eq. 2) between these states is ignored *V*_*E*_ = 0, while right panels correspond to the case when the coupling is included *V*_*E*_ = 0.15 eV. The interference pattern related to the coupling is clearly seeing in the later cases (see arrows).

We focus here on the spectral band corresponding to *ω* − *ω*′ ≈ 13 eV energy loss, which was attributed earlier^21^,^22^,^23^ solely to the 4*σ* → 1*σ* OEOP X-ray emission transition to the final valence state 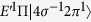, called here the “bright” state. The experimental spectrum shows a three-peak fine structure around *ω* − *ω*′ = 13 eV which evolves with excitation energy *ω* (see Fig. 3). Figure 3a shows that simulations based only on the single *E*′ state result in a single peak envelope which has little resemblance with the experimental spectrum. The reason for this is that the *E*′ potential is crossed by potentials of two “dark” *E*^1^Π 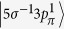 and *G*^1^Π 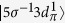 Rydberg states of the same ^1^Π symmetry (Fig. 2), which correspond to TEOP transitions (see Fig. 1). As soon as the coupling of these states with the “bright” *E*′ state, with the corresponding coupling constants *V*_*E*_ and *V*_*G*_ (Eq. 2), is taken into account we obtain excellent agreement with the experiment (Fig. 3a). The simulations of the RIXS profile were performed using (Eqs 3 and 6) extended to the three final states. We emphasize the dynamical aspect of the studied effect which is unraveled by the 2D maps of the nuclear wave packet of the “bright” |*χ*_*E*′_| and “dark” |*χ*_*E*_| states shown in Fig. 4. In order to visualize the dynamics, the “dark” state was artificially populated in our numerical simulations allowing the TEOP transition. The Coulomb mixing (Eq. 2) brings in a new interference pattern into these wave packets. The Fourier transform (Eq. 6) visualizes this pattern in spectral features related to the “dark” E state. The Coulomb coupling thus opens the TEOP decay channels and makes them anomalously strong near the avoided crossing of the potential curves as one can see from Fig. 3a.

Indeed the variation of the excitation energy allows for control of the nuclear wave packet dynamics in the intermediate core excited state and, hence, to control the point of arrival in the final state with respect to the crossing point. This makes RIXS a very powerful tool to study the potential energy surfaces^24^. The *E*′ potential obtained by Lefebvre-Brion^25^ and Guberman^26^ has its bottom at 12.42 eV relative to the ground state minimum, similarly to our *ab initio* calculation. However, this energy position cannot provide a good agreement with the experiment, as it is shown by the dashed lines in Fig. 3. Comparison of the experiment and theory allows for a correction of the position of *E*′'s potential minimum. Fitting the experimental profile (blue lines in Fig. 3) allows to accurately define the minimum of the *E*′ potential to be 12.52 eV.

## Conclusion

Our study illustrates the rich physical content that can be found in the high-resolution RIXS spectra and the possibilities this technique offers to study and even control electron and nuclear quantum dynamics as well as determining precisely the underlying energetics and potential energy surfaces. In case of the CO molecule presented here, this has enabled us to unravel anomalously strong TEOP X-ray decay transitions. The present study also illustrates that advancing spectroscopy techniques may call for a concomitant qualitative advance of the theoretical analysis.

## Methods

### Experimental setup

We measured O K edge RIXS spectra of carbon monoxide using the AERHA spectrometer^27^ mounted at the SEXTANTS beamline^28^ of the SOLEIL light source. For these measurements we have employed the gas cell available at SEXTANTS where CO gas where contained in the cell by mean of a 100 nm thick Si_3_N_4_ membrane. In this way it was possible to measure CO gas at a pressure of 1 bar, while keeping the rest of the experimental setup in UHV (high 10^−8^ mbar), although reducing the transmission by a factor two. In order to minimize the self-absorption effect, we set an angle of 25° between the incident beam and the membrane surface. The scattering angle was 85°. RIXS was measured for both circular and linear polarizations. However, the experimental results does not show significant polarization dependence in an agreement with the calculations and previous experimental study^22^. Due to this we focus here only on the circular polarization while scanning the incoming photon energy across the 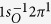 resonance. The combined experimental resolution was 160 meV, the contribution from the beamline bandwidth being 90 meV. The membrane is sufficiently transparent for allowing incoming and scattered soft X-rays to be transmitted but it would easily break under beam exposure. To increase the lifetime we deposited 50 nm of aluminum on both sides of the membrane allowing for days of measurements without ruptures (further reducing the transmission by 15%). On the other hand, aluminum undergoes rapid oxidation giving a non-negligible contribution to the final RIXS spectra during the experiment. In general a contribution from *Al*_2_*O*_3_ emission due to membrane oxidation contaminates the spectra and must be corrected at off-resonance excitation. In the region of interest the contamination implies a weak structureless sloped background only, and the data are presented without any correction.

### Theoretical methods

The potential energy curves for the ground, core excited and final states were computed using the restrict active space self-consistent field (RASSCF) method^29^ followed by second-order perturbation theory (RASPT2) method^30^. The *aug-cc-pVTZ* basis set^31^ was employed, and no symmetry was considered. The transition dipole moments between the core exited and final states were computed with the restricted active space state interaction (RASSI) approach^32^. All calculations were performed with MOLCAS 8.0 software^33^. The lifetime broadening of the core-excited state (2Γ = 0.15 eV) is close to the vibrational frequency (0.18 eV) of this state, which gives rise to lifetime-vibrational interference (LVI) effects^9^. The LVI effect is taken into account explicitly in our wave packet simulations of the RIXS cross section. Some disagreement between the experiment and theory (Fig. 3) is mainly related to the accuracy of the *ab initio* calculations of the diabatic potential energy curves and the coupling constants (Eq. 2). Another reason for the disagreement probably arises from the coupling of the *E*′, *E* and *G* states with the other states neglected in our simulations.

## Additional Information

**How to cite this article**: Couto, R. C. *et al.* Anomalously strong two-electron one-photon X-ray decay transitions in CO caused by avoided crossing. *Sci. Rep.* **6**, 20947; doi: 10.1038/srep20947 (2016).
